# Adipic acid–2,6-bis­(1*H*-benzimidazol-2-yl)pyridine–water (1/2/4)

**DOI:** 10.1107/S1600536812047861

**Published:** 2012-11-28

**Authors:** Songzhu Lin, Ruokun Jia, Feng Gao, Xiaoqing Zhou

**Affiliations:** a Northeast Dianli University, Jilin 132012, People’s Republic of China

## Abstract

The asymmetric unit of the title hydrated co-crystal, 2C_19_H_13_N_5_·C_6_H_10_O_4_·4H_2_O, consists of one 2,6-bis­(1*H*-benzimidazol-2-yl)pyridine mol­ecule, half of an adipic acid mol­ecule (bis­ected by an inversion center) and two water solvates. In the crystal, N—H⋯O, O—H⋯O and O—H⋯N hydrogen bonds and π–π inter­actions [centroid–centroid distances = 3.769 (2) and 3.731 (2) Å] form a three-dimensional supra­molecular structure.

## Related literature

For related structures, see: Boča *et al.* (2000 ▶); Chetia & Iyer (2006 ▶, 2007 ▶); Xiao *et al.* (2010 ▶); Freire *et al.* (2003 ▶); Lin *et al.* (2012 ▶). 
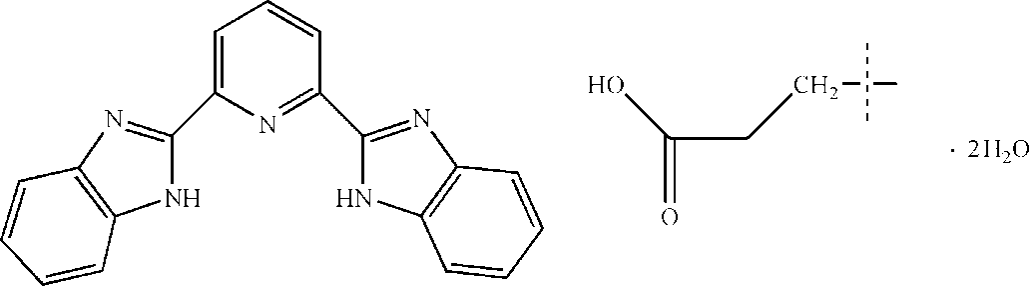


## Experimental

### 

#### Crystal data

2C_19_H_13_N_5_·C_6_H_10_O_4_·4H_2_O
*M* *_r_* = 840.90
Triclinic, 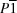

*a* = 9.0709 (18) Å
*b* = 9.6882 (19) Å
*c* = 12.311 (3) Å
α = 88.93 (3)°
β = 83.12 (3)°
γ = 75.14 (3)°
*V* = 1038.1 (4) Å^3^
*Z* = 1
Mo *K*α radiation
μ = 0.10 mm^−1^
*T* = 295 K
0.25 × 0.18 × 0.16 mm

#### Data collection

Enraf–Nonius CAD-4 diffractometer
10214 measured reflections
4705 independent reflections
3555 reflections with *I* > 2σ(*I*)
*R*_int_ = 0.024
3 standard reflections every 100 reflections intensity decay: none

#### Refinement

*R*[*F* ^2^ > 2σ(*F* ^2^)] = 0.047
*wR*(*F* ^2^) = 0.163
*S* = 1.15
4705 reflections
368 parameters
7 restraints
H atoms treated by a mixture of independent and constrained refinement
Δρ_max_ = 0.36 e Å^−3^
Δρ_min_ = −0.19 e Å^−3^

### 

Data collection: *CAD-4 Software* (Enraf–Nonius, 1989 ▶); cell refinement: *CAD-4 Software*; data reduction: *NRCVAX* (Gabe *et al.*, 1989 ▶); program(s) used to solve structure: *SHELXS97* (Sheldrick, 2008 ▶); program(s) used to refine structure: *SHELXL97* (Sheldrick, 2008 ▶); molecular graphics: *SHELXTL* (Sheldrick, 2008 ▶); software used to prepare material for publication: *WinGX* (Farrugia, 2012) ▶.

## Supplementary Material

Crystal structure: contains datablock(s) global, I. DOI: 10.1107/S1600536812047861/bg2486sup1.cif

Structure factors: contains datablock(s) I. DOI: 10.1107/S1600536812047861/bg2486Isup2.hkl

Supplementary material file. DOI: 10.1107/S1600536812047861/bg2486Isup3.cml

Additional supplementary materials:  crystallographic information; 3D view; checkCIF report

## Figures and Tables

**Table 1:** Hydrogen-bond geometry (Å, °)

*D*—H⋯*A*	*D*—H	H⋯*A*	*D*⋯*A*	*D*—H⋯*A*
N2—H4⋯O2*W*	0.95 (2)	2.16 (2)	3.084 (2)	163.3 (18)
N4—H5⋯O2*W*	0.88 (2)	2.08 (2)	2.945 (2)	167.6 (18)
O1—H1⋯N5	0.87 (1)	1.83 (1)	2.6731 (19)	163 (2)
O1*W*—H1*WA*⋯N1^i^	0.85 (1)	1.99 (1)	2.8169 (18)	162 (2)
O1*W*—H1*WB*⋯O2^ii^	0.85 (1)	1.99 (1)	2.815 (2)	165 (2)
O2*W*—H2*WB*⋯O1*W* ^iii^	0.86 (1)	2.05 (1)	2.898 (2)	170 (3)
O2*W*—H2*WA*⋯O1*W* ^iv^	0.87 (1)	2.01 (1)	2.867 (2)	171 (3)

Symmetry codes: (i) 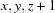; (ii) 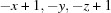; (iii) 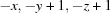; (iv) 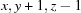.

**Fig. 1.**
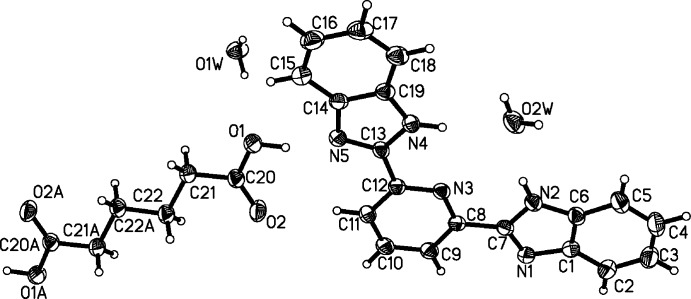
The structure of the title compound showing 30% probability displacement ellipsoids and the atom-numbering scheme.

## supplementary crystallographic information

### Comment

Structures containing 2,6-bis(benzimidazol-2-yl)pyridine have been reported in recent year (Xiao *et al.*, 2010; Freire *et al.*, 2003; Lin *et al.*, 2012). As a continuation of our previous works devoted to structures with 2,6-bis(benzimidazol-2-yl)pyridine, here we report the crystal structures of the title compound (C_19_H_13_N_5_)0.5(C_6_H_10_O_4_)2(H_2_O)(I). Its asymmetric unit consists of one 2-pyridin-4-yl-1*H*-benzoimidazole molecule, half of an adipic acid molecule (bisected by an inversion center) and two water solvates. The aromatic C—C and C—N distances in both the benzimidazole and pyridine rings are within the usual range. All C and N atoms of the 2,6-bis(benzimidazol-2-yl)pyridine molecule lay in a plane (largest deviation: 0.084 Å for C16). The compound is similar to other related compounds consisting of 2,6-bis(benzimidazol-2-yl)pyridine and organic molecules (Boča *et al.*, 2000; Chetia and Iyer, 2006; Chetia and Iyer, 2007; Xiao *et al.*, 2010; Lin *et al.*, 2012).

The crystal structure in (I) is a 3D array, definend by N—H···O, O—H···O, O—H···N inter- and intramolecular interactions (Table 1) and π–π stacking interactions between rings with center-to-center distances *Cg*1···*Cg*2 = 3.769 (2) (symmetry code 1 - *x*, 1 - *y*, -*z*), *Cg*2···*Cg*3=3.731 (2) Å (symmetry code -*x*, 1 - *y*, -*z*), where *Cg*1, *Cg*2 and *Cg*3 refer to imidazole ring N4—C13—N5—C14—C19, pyridine ring N3—C8—C9—C10—C11—C12 and phenyl ring C1—C2—C3—C4—C5—C6, respectively.

### Experimental

The title compound was obtained by 2,6-bis(benzimidazol-2-yl)pyridine (0.062 g, 0.20 mmol) and adipic acid (0.029 g, 0.20 mmol) dissolved in 30 ml solution mixed with ethanol and water by 2:1(V/V) was heated to refluxed for 8 h and cooled to the room temperature. Single crystals suitable for X-ray measurements were obtained by recrystallization at room temperature.

### Refinement

The positions of all H atoms were found in a difference Fourier map, and refined both in coordinates as in displacement factors. Those attached to O were subject to distance restraints (O-H = 0.85 (1)Å).

### Crystal data

**Table:**

2C_19_H_13_N_5_·C_6_H_10_O_4_·4H_2_O	*Z* = 1
*M**_r_* = 840.90	*F*(000) = 442
Triclinic, *P*1	*D*_x_ = 1.345 Mg m^−^^3^
Hall symbol: -P 1	Mo *K*α radiation, λ = 0.71073 Å
*a* = 9.0709 (18) Å	Cell parameters from 25 reflections
*b* = 9.6882 (19) Å	θ = 4–14°
*c* = 12.311 (3) Å	µ = 0.10 mm^−^^1^
α = 88.93 (3)°	*T* = 295 K
β = 83.12 (3)°	Block, yellow
γ = 75.14 (3)°	0.25 × 0.18 × 0.16 mm
*V* = 1038.1 (4) Å^3^	

### Data collection

**Table:**

Enraf–Nonius CAD-4 diffractometer	*R*_int_ = 0.024
Radiation source: fine-focus sealed tube	θ_max_ = 27.5°, θ_min_ = 3.0°
Graphite monochromator	*h* = −11→11
ω scans	*k* = −12→12
10214 measured reflections	*l* = −15→15
4705 independent reflections	3 standard reflections every 100 reflections
3555 reflections with *I* > 2σ(*I*)	intensity decay: none

### Refinement

**Table:**

Refinement on *F*^2^	Primary atom site location: structure-invariant direct methods
Least-squares matrix: full	Secondary atom site location: difference Fourier map
*R*[*F*^2^ > 2σ(*F*^2^)] = 0.047	Hydrogen site location: inferred from neighbouring sites
*wR*(*F*^2^) = 0.163	H atoms treated by a mixture of independent and constrained refinement
*S* = 1.15	*w* = 1/[σ^2^(*F*_o_^2^) + (0.1*P*)^2^ + 0.0268*P*] where *P* = (*F*_o_^2^ + 2*F*_c_^2^)/3
4705 reflections	(Δ/σ)_max_ < 0.001
368 parameters	Δρ_max_ = 0.36 e Å^−^^3^
7 restraints	Δρ_min_ = −0.19 e Å^−^^3^

### Special details

**Table:**

Geometry. All esds (except the esd in the dihedral angle between two l.s. planes) are estimated using the full covariance matrix. The cell esds are taken into account individually in the estimation of esds in distances, angles and torsion angles; correlations between esds in cell parameters are only used when they are defined by crystal symmetry. An approximate (isotropic) treatment of cell esds is used for estimating esds involving l.s. planes.
Refinement. Refinement of F^2^ against ALL reflections. The weighted R-factor wR and goodness of fit S are based on F^2^, conventional R-factors R are based on F, with F set to zero for negative F^2^. The threshold expression of F^2^ > 2sigma(F^2^) is used only for calculating R-factors(gt) etc. and is not relevant to the choice of reflections for refinement. R-factors based on F^2^ are statistically about twice as large as those based on F, and R- factors based on ALL data will be even larger.

### Fractional atomic coordinates and isotropic or equivalent isotropic displacement parameters (Å^2^)

**Table:**

	*x*	*y*	*z*	*U*_iso_*/*U*_eq_	
N1	0.13159 (14)	0.33800 (13)	−0.15941 (10)	0.0488 (3)	
N2	0.11534 (14)	0.56273 (13)	−0.10937 (10)	0.0469 (3)	
H4	0.142 (2)	0.632 (2)	−0.0672 (17)	0.070 (5)*	
N3	0.30720 (13)	0.47411 (12)	0.04908 (9)	0.0425 (3)	
N4	0.35862 (15)	0.69020 (12)	0.16663 (10)	0.0461 (3)	
H5	0.296 (2)	0.718 (2)	0.1169 (17)	0.071 (6)*	
N5	0.51881 (14)	0.53946 (12)	0.26517 (10)	0.0467 (3)	
C1	0.03749 (17)	0.43165 (17)	−0.22421 (12)	0.0502 (3)	
C2	−0.0392 (2)	0.4018 (2)	−0.30889 (15)	0.0660 (5)	
H2B	−0.029 (3)	0.302 (3)	−0.331 (2)	0.108 (9)*	
C3	−0.1263 (2)	0.5167 (3)	−0.36062 (17)	0.0737 (5)	
H3B	−0.177 (2)	0.500 (2)	−0.4221 (17)	0.075 (6)*	
C4	−0.1367 (2)	0.6559 (3)	−0.32926 (17)	0.0738 (6)	
H4B	−0.203 (2)	0.737 (2)	−0.3704 (18)	0.083 (6)*	
C5	−0.0618 (2)	0.6887 (2)	−0.24504 (16)	0.0625 (4)	
H5B	−0.071 (2)	0.784 (2)	−0.2255 (17)	0.072 (6)*	
C6	0.02658 (16)	0.57252 (17)	−0.19362 (12)	0.0495 (4)	
C7	0.17580 (15)	0.42054 (14)	−0.09313 (11)	0.0436 (3)	
C8	0.28186 (15)	0.37145 (14)	−0.01174 (11)	0.0423 (3)	
C9	0.35464 (18)	0.22746 (15)	−0.00130 (13)	0.0500 (4)	
H9A	0.3296 (19)	0.1624 (19)	−0.0465 (15)	0.053 (4)*	
C10	0.45920 (19)	0.18978 (15)	0.07321 (13)	0.0533 (4)	
H10A	0.517 (2)	0.086 (2)	0.0801 (15)	0.064 (5)*	
C11	0.48672 (19)	0.29346 (15)	0.13676 (13)	0.0496 (4)	
H11A	0.553 (2)	0.2771 (19)	0.1892 (15)	0.058 (5)*	
C12	0.40660 (15)	0.43502 (14)	0.12290 (11)	0.0423 (3)	
C13	0.42912 (15)	0.55179 (14)	0.18674 (11)	0.0424 (3)	
C14	0.50736 (17)	0.67897 (15)	0.29760 (12)	0.0461 (3)	
C15	0.5824 (2)	0.72962 (19)	0.37449 (15)	0.0590 (4)	
H15A	0.653 (3)	0.659 (2)	0.4172 (19)	0.085 (6)*	
C16	0.5557 (2)	0.8759 (2)	0.38559 (16)	0.0678 (5)	
H16A	0.607 (2)	0.915 (2)	0.4359 (18)	0.080 (6)*	
C17	0.4553 (2)	0.96933 (19)	0.32354 (16)	0.0686 (5)	
H17A	0.442 (2)	1.074 (2)	0.3320 (17)	0.080 (6)*	
C18	0.3784 (2)	0.92146 (17)	0.24903 (14)	0.0588 (4)	
H18A	0.308 (2)	0.984 (2)	0.2049 (17)	0.072 (6)*	
C19	0.40766 (17)	0.77399 (15)	0.23620 (12)	0.0465 (3)	
O1	0.67965 (16)	0.37143 (12)	0.40769 (11)	0.0688 (4)	
H1	0.630 (2)	0.411 (2)	0.3538 (14)	0.084 (7)*	
O2	0.74254 (17)	0.17221 (13)	0.30895 (11)	0.0732 (4)	
C20	0.75243 (17)	0.23700 (16)	0.38907 (13)	0.0491 (3)	
C21	0.8489 (2)	0.17588 (17)	0.47764 (14)	0.0538 (4)	
H21A	0.784 (3)	0.179 (2)	0.5432 (19)	0.079 (6)*	
H21B	0.915 (2)	0.250 (2)	0.4912 (16)	0.073 (6)*	
C22	0.9496 (2)	0.02613 (17)	0.45481 (14)	0.0543 (4)	
H22A	0.883 (2)	−0.040 (2)	0.4464 (16)	0.069 (5)*	
H22B	1.019 (2)	0.022 (2)	0.3830 (17)	0.071 (5)*	
O1W	0.14244 (18)	0.04491 (14)	0.85092 (11)	0.0742 (4)	
O2W	0.14234 (16)	0.82851 (14)	0.01294 (13)	0.0751 (4)	
H1WA	0.153 (3)	0.1282 (13)	0.8350 (18)	0.102 (8)*	
H1WB	0.172 (3)	−0.0080 (19)	0.7943 (13)	0.093 (8)*	
H2WB	0.064 (3)	0.870 (3)	0.058 (2)	0.152 (13)*	
H2WA	0.151 (3)	0.896 (2)	−0.0326 (18)	0.126 (10)*	

### Atomic displacement parameters (Å^2^)

**Table:**

	*U*^11^	*U*^22^	*U*^33^	*U*^12^	*U*^13^	*U*^23^
N1	0.0533 (7)	0.0439 (7)	0.0461 (7)	−0.0062 (5)	−0.0072 (5)	−0.0025 (5)
N2	0.0474 (6)	0.0400 (6)	0.0505 (7)	−0.0060 (5)	−0.0065 (5)	0.0026 (5)
N3	0.0460 (6)	0.0359 (5)	0.0420 (6)	−0.0054 (5)	−0.0027 (5)	0.0022 (4)
N4	0.0533 (7)	0.0361 (6)	0.0453 (7)	−0.0042 (5)	−0.0081 (5)	0.0051 (5)
N5	0.0524 (7)	0.0387 (6)	0.0450 (6)	−0.0034 (5)	−0.0080 (5)	−0.0001 (5)
C1	0.0489 (8)	0.0545 (8)	0.0434 (8)	−0.0071 (7)	−0.0036 (6)	−0.0008 (6)
C2	0.0642 (10)	0.0800 (13)	0.0525 (10)	−0.0130 (9)	−0.0134 (8)	−0.0017 (9)
C3	0.0645 (11)	0.0970 (15)	0.0574 (11)	−0.0116 (10)	−0.0188 (9)	0.0053 (10)
C4	0.0572 (10)	0.0880 (14)	0.0688 (12)	−0.0038 (10)	−0.0142 (9)	0.0243 (11)
C5	0.0539 (9)	0.0595 (10)	0.0681 (11)	−0.0048 (8)	−0.0074 (8)	0.0158 (8)
C6	0.0429 (7)	0.0524 (8)	0.0491 (8)	−0.0068 (6)	−0.0025 (6)	0.0079 (6)
C7	0.0452 (7)	0.0374 (7)	0.0438 (7)	−0.0047 (6)	−0.0005 (6)	0.0003 (5)
C8	0.0449 (7)	0.0379 (7)	0.0403 (7)	−0.0060 (6)	−0.0005 (5)	0.0001 (5)
C9	0.0589 (8)	0.0353 (7)	0.0522 (8)	−0.0058 (6)	−0.0058 (7)	−0.0029 (6)
C10	0.0638 (9)	0.0345 (7)	0.0552 (9)	−0.0002 (7)	−0.0097 (7)	0.0018 (6)
C11	0.0568 (8)	0.0379 (7)	0.0498 (8)	−0.0027 (6)	−0.0110 (7)	0.0047 (6)
C12	0.0461 (7)	0.0363 (6)	0.0410 (7)	−0.0056 (6)	−0.0025 (5)	0.0021 (5)
C13	0.0451 (7)	0.0354 (6)	0.0424 (7)	−0.0034 (5)	−0.0038 (5)	0.0042 (5)
C14	0.0520 (8)	0.0401 (7)	0.0423 (7)	−0.0064 (6)	−0.0018 (6)	−0.0031 (6)
C15	0.0625 (9)	0.0558 (9)	0.0554 (9)	−0.0064 (8)	−0.0111 (7)	−0.0087 (7)
C16	0.0759 (11)	0.0624 (10)	0.0662 (11)	−0.0187 (9)	−0.0073 (9)	−0.0187 (9)
C17	0.0898 (13)	0.0429 (8)	0.0714 (12)	−0.0168 (9)	−0.0015 (10)	−0.0101 (8)
C18	0.0761 (11)	0.0375 (7)	0.0587 (10)	−0.0090 (7)	−0.0035 (8)	0.0002 (7)
C19	0.0543 (8)	0.0387 (7)	0.0430 (7)	−0.0078 (6)	−0.0006 (6)	0.0009 (6)
O1	0.0873 (9)	0.0462 (6)	0.0612 (7)	0.0133 (6)	−0.0288 (7)	−0.0021 (5)
O2	0.0936 (9)	0.0482 (6)	0.0773 (8)	0.0008 (6)	−0.0494 (7)	−0.0055 (6)
C20	0.0528 (8)	0.0410 (7)	0.0520 (8)	−0.0055 (6)	−0.0152 (6)	0.0062 (6)
C21	0.0598 (9)	0.0492 (8)	0.0475 (9)	−0.0009 (7)	−0.0154 (7)	0.0048 (6)
C22	0.0600 (9)	0.0463 (8)	0.0543 (9)	−0.0033 (7)	−0.0209 (8)	0.0062 (7)
O1W	0.1059 (10)	0.0511 (7)	0.0632 (8)	−0.0195 (7)	−0.0014 (7)	−0.0007 (6)
O2W	0.0710 (8)	0.0589 (7)	0.0809 (10)	0.0058 (6)	−0.0037 (7)	0.0200 (7)

### Geometric parameters (Å, º)

**Table:**

N1—C7	1.3166 (19)	C11—C12	1.3957 (19)
N1—C1	1.388 (2)	C11—H11A	0.917 (19)
N2—C7	1.3664 (17)	C12—C13	1.459 (2)
N2—C6	1.375 (2)	C14—C15	1.394 (2)
N2—H4	0.95 (2)	C14—C19	1.395 (2)
N3—C8	1.3375 (18)	C15—C16	1.381 (3)
N3—C12	1.3381 (19)	C15—H15A	1.00 (2)
N4—C13	1.3617 (17)	C16—C17	1.397 (3)
N4—C19	1.376 (2)	C16—H16A	0.96 (2)
N4—H5	0.88 (2)	C17—C18	1.370 (3)
N5—C13	1.3201 (18)	C17—H17A	1.00 (2)
N5—C14	1.3911 (18)	C18—C19	1.393 (2)
C1—C2	1.394 (2)	C18—H18A	0.98 (2)
C1—C6	1.398 (2)	O1—C20	1.3109 (19)
C2—C3	1.383 (3)	O1—H1	0.873 (10)
C2—H2B	0.99 (3)	O2—C20	1.203 (2)
C3—C4	1.386 (3)	C20—C21	1.498 (2)
C3—H3B	0.97 (2)	C21—C22	1.514 (2)
C4—C5	1.388 (3)	C21—H21A	0.94 (2)
C4—H4B	1.03 (2)	C21—H21B	1.07 (2)
C5—C6	1.394 (2)	C22—C22^i^	1.522 (3)
C5—H5B	0.94 (2)	C22—H22A	1.001 (19)
C7—C8	1.461 (2)	C22—H22B	1.02 (2)
C8—C9	1.3927 (19)	O1W—H1WA	0.854 (10)
C9—C10	1.376 (2)	O1W—H1WB	0.848 (9)
C9—H9A	0.935 (18)	O2W—H2WB	0.860 (10)
C10—C11	1.374 (2)	O2W—H2WA	0.865 (10)
C10—H10A	1.012 (18)		
			
C7—N1—C1	104.88 (12)	N3—C12—C11	122.93 (14)
C7—N2—C6	106.77 (13)	N3—C12—C13	115.23 (12)
C7—N2—H4	120.0 (12)	C11—C12—C13	121.82 (14)
C6—N2—H4	133.2 (12)	N5—C13—N4	112.69 (13)
C8—N3—C12	117.85 (12)	N5—C13—C12	126.24 (12)
C13—N4—C19	107.10 (13)	N4—C13—C12	121.05 (13)
C13—N4—H5	125.1 (13)	N5—C14—C15	129.82 (15)
C19—N4—H5	127.8 (13)	N5—C14—C19	109.63 (13)
C13—N5—C14	105.00 (12)	C15—C14—C19	120.50 (15)
N1—C1—C2	129.23 (17)	C16—C15—C14	117.41 (17)
N1—C1—C6	109.80 (14)	C16—C15—H15A	124.0 (13)
C2—C1—C6	120.96 (16)	C14—C15—H15A	118.6 (13)
C3—C2—C1	117.3 (2)	C15—C16—C17	121.28 (17)
C3—C2—H2B	122.5 (16)	C15—C16—H16A	120.3 (13)
C1—C2—H2B	120.2 (16)	C17—C16—H16A	118.4 (13)
C2—C3—C4	121.2 (2)	C18—C17—C16	122.09 (16)
C2—C3—H3B	119.3 (13)	C18—C17—H17A	119.2 (12)
C4—C3—H3B	119.5 (13)	C16—C17—H17A	118.7 (12)
C3—C4—C5	122.70 (18)	C17—C18—C19	116.66 (17)
C3—C4—H4B	117.8 (13)	C17—C18—H18A	123.8 (12)
C5—C4—H4B	119.5 (13)	C19—C18—H18A	119.6 (12)
C4—C5—C6	115.93 (19)	N4—C19—C18	132.36 (15)
C4—C5—H5B	121.0 (13)	N4—C19—C14	105.57 (12)
C6—C5—H5B	123.1 (13)	C18—C19—C14	122.03 (15)
N2—C6—C5	132.52 (16)	C20—O1—H1	113.3 (16)
N2—C6—C1	105.55 (13)	O2—C20—O1	123.00 (14)
C5—C6—C1	121.93 (17)	O2—C20—C21	124.71 (14)
N1—C7—N2	112.99 (13)	O1—C20—C21	112.28 (14)
N1—C7—C8	125.67 (12)	C20—C21—C22	114.80 (14)
N2—C7—C8	121.32 (13)	C20—C21—H21A	108.3 (13)
N3—C8—C9	122.74 (14)	C22—C21—H21A	110.4 (13)
N3—C8—C7	115.52 (12)	C20—C21—H21B	106.6 (10)
C9—C8—C7	121.71 (13)	C22—C21—H21B	111.9 (11)
C10—C9—C8	118.51 (14)	H21A—C21—H21B	104.2 (17)
C10—C9—H9A	124.2 (11)	C21—C22—C22^i^	111.97 (18)
C8—C9—H9A	117.3 (11)	C21—C22—H22A	109.3 (11)
C11—C10—C9	119.71 (13)	C22^i^—C22—H22A	109.8 (11)
C11—C10—H10A	120.1 (10)	C21—C22—H22B	110.5 (11)
C9—C10—H10A	120.2 (10)	C22^i^—C22—H22B	108.1 (11)
C10—C11—C12	118.22 (14)	H22A—C22—H22B	107.0 (15)
C10—C11—H11A	125.1 (11)	H1WA—O1W—H1WB	108.7 (18)
C12—C11—H11A	116.6 (11)	H2WB—O2W—H2WA	102.4 (18)

Symmetry code: (i) −*x*+2, −*y*, −*z*+1.

### Hydrogen-bond geometry (Å, º)

**Table:**

*D*—H···*A*	*D*—H	H···*A*	*D*···*A*	*D*—H···*A*
N2—H4···O2*W*	0.95 (2)	2.16 (2)	3.084 (2)	163.3 (18)
N4—H5···O2*W*	0.88 (2)	2.08 (2)	2.945 (2)	167.6 (18)
O1—H1···N5	0.87 (1)	1.83 (1)	2.6731 (19)	163 (2)
O1*W*—H1*WA*···N1^ii^	0.85 (1)	1.99 (1)	2.8169 (18)	162 (2)
O1*W*—H1*WB*···O2^iii^	0.85 (1)	1.99 (1)	2.815 (2)	165 (2)
O2*W*—H2*WB*···O1*W*^iv^	0.86 (1)	2.05 (1)	2.898 (2)	170 (3)
O2*W*—H2*WA*···O1*W*^v^	0.87 (1)	2.01 (1)	2.867 (2)	171 (3)

Symmetry codes: (ii) *x*, *y*, *z*+1; (iii) −*x*+1, −*y*, −*z*+1; (iv) −*x*, −*y*+1, −*z*+1; (v) *x*, *y*+1, *z*−1.
